# *In silico *studies of natural product-like caffeine derivatives as potential MAO-B inhibitors/AA_2A_R antagonists for the treatment of Parkinson's disease

**DOI:** 10.1515/jib-2021-0027

**Published:** 2022-09-19

**Authors:** Yassir Boulaamane, Mahmoud A. A. Ibrahim, Mohammed Reda Britel, Amal Maurady

**Affiliations:** Laboratory of Innovative Technologies, National School of Applied Sciences of Tangier, Abdelmalek Essaadi University, Tetouan, Morocco; Computational Chemistry Laboratory, Chemistry Department, Faculty of Science, Minia University, Minia, 61519, Egypt; Faculty of Sciences and Techniques of Tangier, Abdelmalek Essaadi University, Tetouan, Morocco

**Keywords:** ADMET prediction, caffeine, molecular dynamics simulation, natural products, neuroprotection, structure-based virtual screening

## Abstract

Parkinson’s disease is considered the second most frequent neurodegenerative disease. It is described by the loss of dopaminergic neurons in the mid-brain. For many decades, L-DOPA has been considered as the gold standard for treating Parkinson’s disease motor symptoms, however, due to the decrease of efficacy, in the long run, there is an urgent need for novel antiparkinsonian drugs. Caffeine derivatives have been reported several times for their neuroprotective properties and dual blockade of monoamine oxidase (MAO) and adenosine A_2A_ receptors (AA_2A_R). Natural products are currently attracting more focus due to structural diversity and safety in contrast to synthetic drugs. In the present work, computational studies were conducted on natural product-like caffeine derivatives to search for novel potent candidates acting as dual MAO-B inhibitors/AA_2A_R antagonists for Parkinson’s disease. Our findings revealed two natural products among the top hits: CNP0202316 and CNP0365210 fulfill the requirements of drugs acting on the brain. The selected lead compounds were further studied using molecular dynamics simulation to assess their stability with MAO-B. Current findings might shift the interest towards natural-based compounds and could be exploited to further optimize caffeine derivatives into a successful dual-target-directed drug for managing and halting the neuronal damage in Parkinson’s disease patients.

## 1 Introduction

Neurodegenerative diseases and brain-associated diseases are major concerns among aging populations across the world [1]. Neurodegenerative diseases such as Parkinson’s and Alzheimer’s diseases have a multifactorial nature that is characterized by the progressive loss of neurons in the brain [2]. Parkinson’s disease (PD) is defined especially by the progressive loss of dopaminergic neurons in the substantia nigra pars compacta (SNpc) of the midbrain [3]. More than six million people in the world are affected today with a prevalence of 150 in every 100,000 people which is further increasing with age and affects 1% of the population over 60 years [4]. Current pharmaceutical treatments for PD include levodopa or levodopa plus dopa-decarboxylase inhibitors, dopamine agonists, and catechol-O-methyl transferase (COMT)/monoamine oxidase B (MAO-B) inhibitors [5]. Recently, other non-dopaminergic drugs have shown promising efficacy to relieve PD symptoms such as adenosine A_2A_ receptor (AA_2A_R) antagonists [6].

Monoamine Oxidase (MAO) (EC 1.4.3.4) belongs to a family of flavin adenine dinucleotide (FAD)-dependant enzymes that are expressed in the outer mitochondrial membrane of neuronal cells. The MAO enzymes are responsible for the oxidative deamination of monoamine neurotransmitters such as dopamine, adrenaline, and noradrenaline in the central nervous system (CNS) [5, 7]. The MAO enzymes exist in two isoforms, MAO-A and MAO-B that share sequence similarities of 70% but differ in tissue distribution, substrate, and inhibitor preferences [5]. The development of the first MAO inhibitors was abandoned due to side effects related to the metabolism of tyramine, which causes a cardiovascular crisis [8]. However, a new class of selective MAO-B inhibitors has been proven to be efficient in treating PD symptoms. It was also shown that this new class of selective MAO-B inhibitors is devoid of tyramine-related side effects. Furthermore, the selective MAO-B inhibitors may act as neuroprotective agents by limiting the release of free radical species and hence may decrease the progression of the disease [5, 9].

MAO-A preferentially metabolizes serotonin while MAO-B preferentially deaminates 2-phenylethylamine and benzylamine. Dopamine, norepinephrine, and epinephrine are substrates of both isoforms in most animal tissues [10].

During aging, the expression of MAO-B increases in the brain and is connected with an enhanced dopamine metabolism which results in an increased reactive oxygen species (ROS) production such as hydrogen peroxide (H_2_O_2_) inducing oxidative damage and apoptotic signaling events [11].

**Figure 1:**
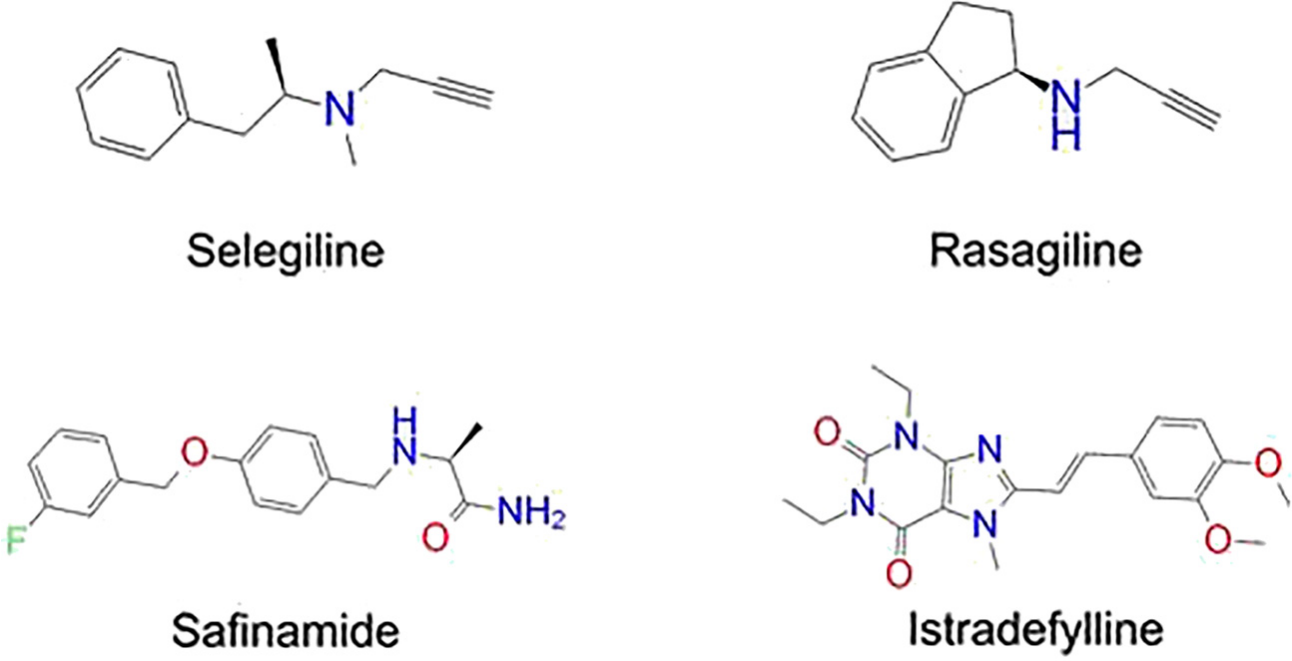
Chemical structures of monoamine oxidase B inhibitors.

Previously approved MAO-B inhibitors are selegiline and rasagiline which irreversibly inhibit MAO-B with an IC_50_ value of 6.8 and 14 nM respectively [12]. The latest approved MAO-B inhibitor is safinamide which reversibly inhibits MAO-B with an IC_50_ value of 450 nM [13]. Istradefylline, a caffeine-based inhibitor that was approved in Japan in 2013 and also approved for medical use in the United States in 2019 acts as a dual inhibitor of MAO-B and AA_2A_R [14, 15]. However, istradefylline was found to be a weak inhibitor of MAO-B (IC_50_ = 28 μM) which encourages further research on new substitutions to the caffeine core [16]. The chemical structures of MAO-B inhibitors are shown in Figure 1.

**Figure 2:**
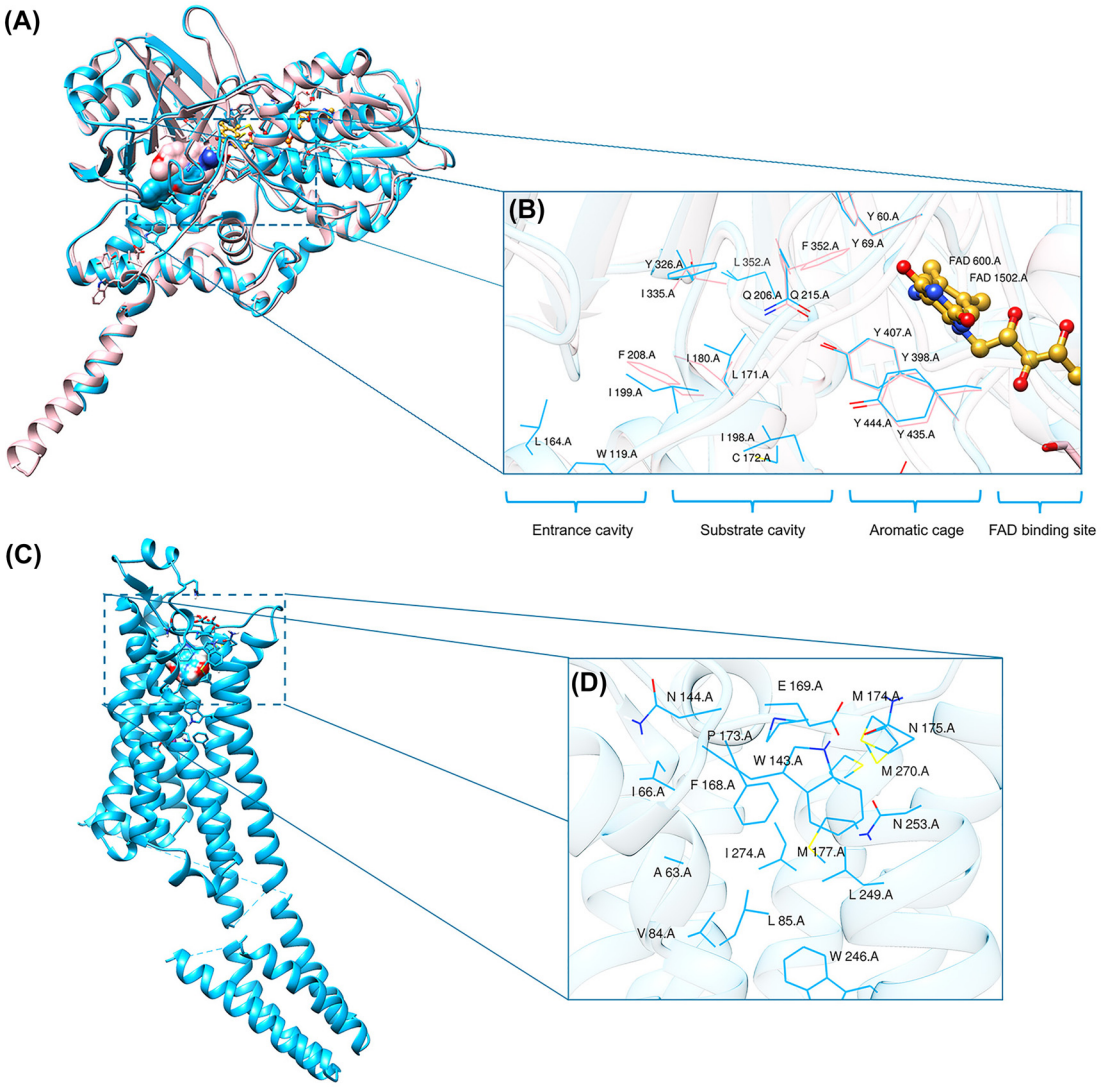
(A) Superposition of crystal structures of MAO-A (pink color) and MAO-B (deep sky-blue color). (B) Superposition of active site residues of MAO-A (pink color) and MAO-B (deep sky-blue color), FAD (goldenrod color) is shown in ball & stick representation. (C) Crystal structure of AA_2A_R in complex with caffeine. (D) Active site residues of AA_2A_R.

The crystal structure of MAO-A (PDB ID: 2Z5Y) has a monopartite substrate cavity of ∼550 Å^3^ volume while the crystal structure of MAO-B contains a bipartite cavity structure with an entrance cavity of ∼290 Å^3^ and a substrate cavity of ∼400 Å^3^ [17]. ILE-199 and TYR-326 separate these two cavities in MAO-B serving as “gating” residues and a structural determinant for substrate and inhibitor recognition by MAO-B [18, 19]. The superposition of MAO-A and MAO-B and their active site residues are shown in Figure 2.

The structural study revealed that MAO-B (PDB ID: 2V5Z) is formed by two monomers consisting of a globular domain anchored to the membrane through a C-terminal helix [20]. MAO-B active site residues that share similarities to MAO-A active site are TYR-60, LEU-164, PHE-168, GLN-206, ILE-198, ILE-316, PHE-343, TYR-398, and TYR-435. Meanwhile, the amino acids that are specific to MAO-B are located in the hydrophobic pocket which is formed by LEU-171, CYS-172, ILE-199, TYR-326 [13].

There is a great deal of literature supporting the use and efficacy of natural products (NP) in PD such as flavonoids, xanthones, phenolic derivatives, alkaloids, and caffeine [21, 22]. These natural resources and their derivatives have been reported for their potential to selectively inhibit MAO-B and may offer a safer alternative compared to conventional drugs [23]. Furthermore, caffeine has been used in several studies as a scaffold for the design of dual MAO inhibitors/AA_2A_R antagonists. Pretorius et al. synthesized a series of C-8 substituted caffeinyl analogues and it was found that the compound bearing a 4-phenylbutadien moiety is the most potent candidate for MAO-B and AA_2A_R [24]. On the other hand, Azam et al. explored numerous caffeine derivatives from the literature bearing multiple substitutions through molecular docking and structure-activity relationship studies, it was found that the placement of hydrophobic moieties at C8 is essential for both MAO-B inhibition and AA_2A_R antagonism, whereas replacements occurring at C1 and C3 are optimal for AA_2A_R but not detrimental for MAO-B [25]. Although research on caffeine is underway for decades, its naturally occurring derivatives are yet to be investigated in detail [26].

NPs and NP-based compounds are an ideal choice for scientists and researchers due to the broad-spectrum activity of NPs with their minimal or no toxic effect on human health [27]. The literature has indicated that caffeine among other NPs is a potent compound that has neuroprotective properties [28]. Considering the link between neurodegeneration and oxidative stress due to the mitochondrial imbalance and the accumulation of reactive oxygen species (ROS), MAO-B was and still is, considered a valid therapeutic target for slowing down the progression of Parkinson’s disease.

In the present study, a substructure search was conducted on natural products databases to retrieve caffeine-containing natural products since it is known for its neuroprotective properties and its potency to act as an antagonist of AA_2A_R, a validated target for PD [29]. Structure-based virtual screening was employed to evaluate the affinity of the selected natural compounds towards MAO-B and AA_2A_R. ADMET properties were evaluated using *in silico* methods. Finally, molecular dynamics simulations were performed to study the interactions and the stability between the selected compounds and MAO-B over the simulation time.

## 2 Material and methods

### 2.1 Data sources

To retrieve all the available natural compounds based on the caffeine scaffold, we used the COCONUT database (https://coconut.naturalproducts.net/); the largest open-source natural products (NP) database to date containing more than 400 000 unique NP from over 50 sources [30]. The search was conducted using the Ulmann algorithm for the substructure search with the caffeine scaffold as a pharmacophore [31]. The search results revealed 144 caffeine-containing natural products. These compounds were downloaded in SDF format for further analysis.

### 2.2 Protein preparation and grid generation

Crystal structure of MAO-B (PDB ID: 2V5Z, resolution = 1.7 Å) in complex with safinamide and crystal structure of AA_2A_R (PDB ID: 5MZP, resolution = 2.1 Å) in complex with caffeine were retrieved from the RCSB Protein Data Bank (https://www.rcsb.org/) [13]. Residues with missing atoms were fixed using the CHARMM-GUI web server [32]. Water molecules were removed since they are not involved in the ligand binding. Since MAO-B is expressed as a dimer, only one chain was kept along with the FAD cofactor for the molecular modeling studies to ease the computational cost [33]. Finally, polar hydrogens and Kollman charges were added using AutoDockTools 1.5.6 [34]. The grid box was placed near the FAD cofactor with a spacing of 1 Å. Grid dimensions were chosen large enough (24 × 24 × 24 Å in x, y, and z directions, respectively) to fit all the residues forming both cavities of the active site in the protein. The grid box was positioned in a way to cover the entire binding site and to allow larger molecules to dock properly: 53 × 155 × 27 Å for MAO-B and −21.6 × 6.1 × 17.5 for AA_2A_R in *x*, *y*, and *z* directions, respectively. Lastly, the generated coordinates for the grid box were saved in a text file.

### 2.3 Preparation of ligands

The selected caffeine derivatives were split into multiple files, with each file containing a single ligand. The 3D conformations were generated for all the compounds, geometrical optimization was performed using Merck molecular force field (MMFF94) implemented in the Open Babel chemical toolbox [35]. The minimized ligands were then prepared for the molecular docking study using the prepare_ligand4.py package of AutoDockTools 1.5.6. Partial charges, atomic types, and polar hydrogens were added to all compounds and then converted to PDBQT format.

### 2.4 Structure-based virtual screening workflow

**Figure 3:**
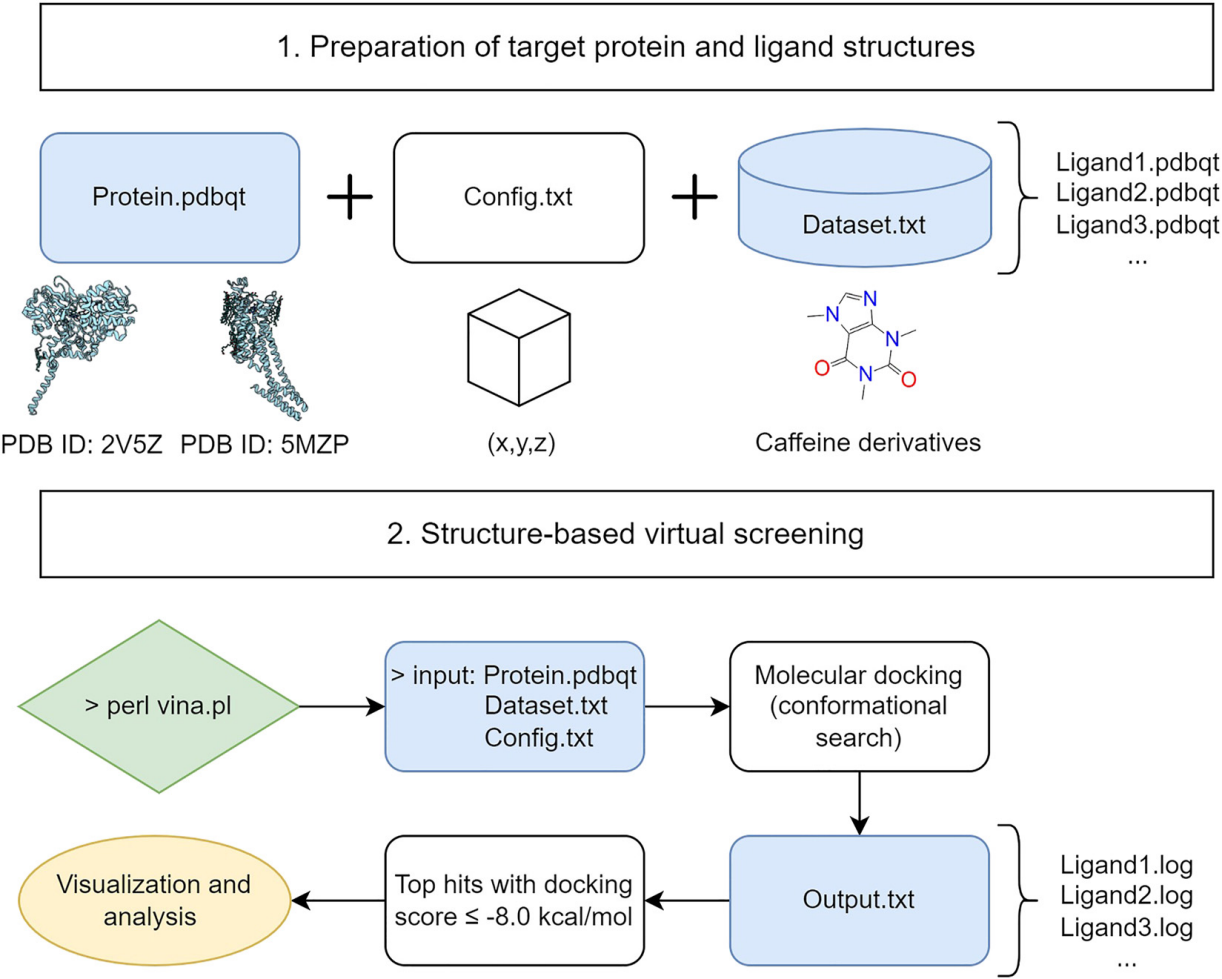
Proposed workflow for structure-based virtual screening using AutoDock Vina.

**Figure 4:**
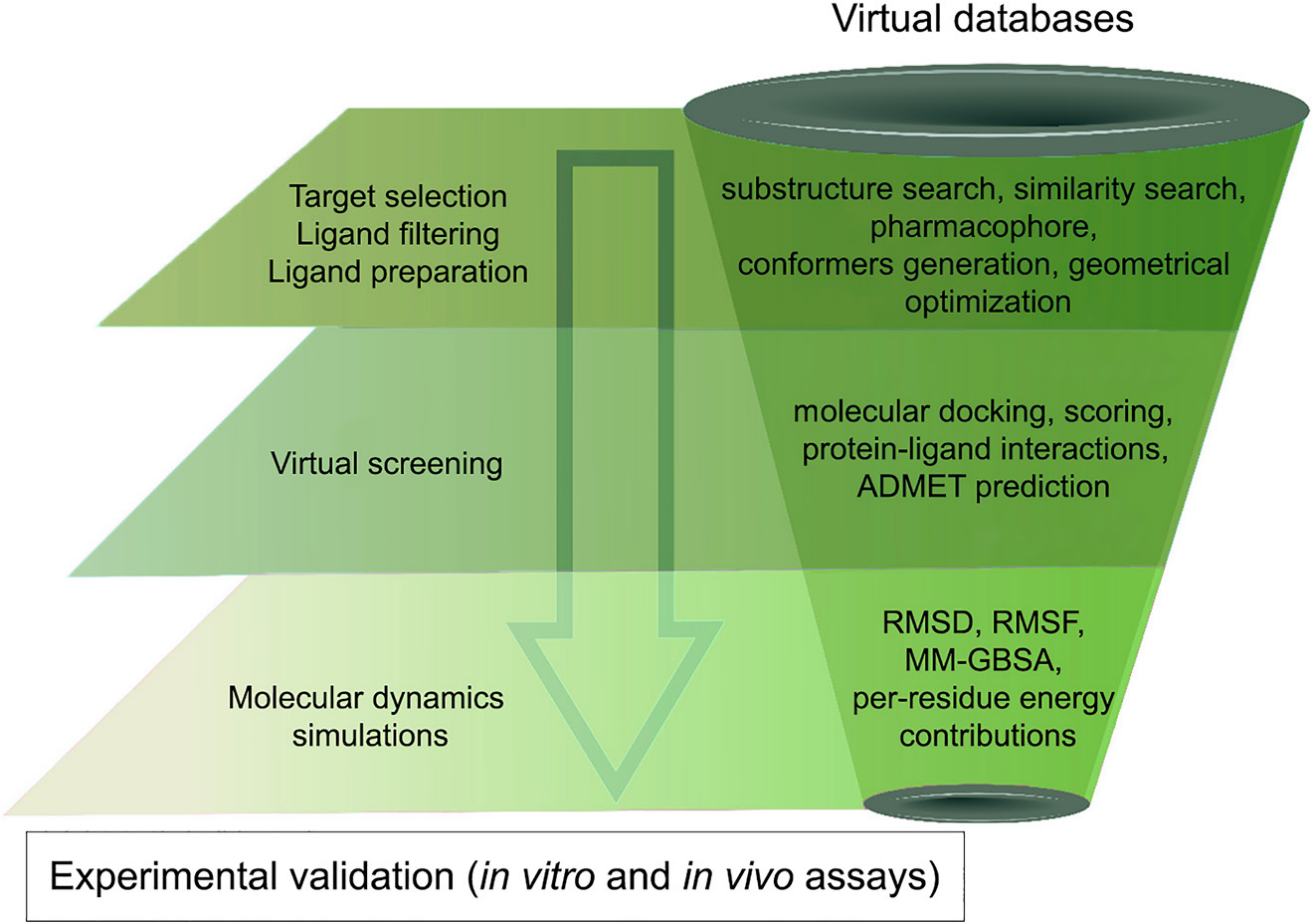
State-of-the-art virtual screening methodology to select and study natural products.

Structure-based virtual screening was performed using a Perl script for the automated execution of AutoDock Vina 1.1.2 [36]. The proposed methodology is detailed in Figure 3, a text file containing all the names of the prepared ligands was created to serve as a single input file for the docking screens. To facilitate the analysis of the virtual screening results, all the generated output log files were concatenated into a single output text file. All procedures were performed in respect of good practices using state-of-the-art virtual screening approaches for natural products bioprospecting as shown in Figure 4. The standard virtual screening consists mainly of target identification, selection of the chemical library, molecular docking studies, ADMET evaluation, molecular dynamics simulations, and finally experimental validation of the lead compounds [37].

### 2.5 Visualization and analysis

The ligands were ranked by their binding affinities. The compounds displaying a binding score of −8.0 kcal/mol or less were subject to further analysis. The conformations of the selected compounds were visualized using UCSF Chimera visualization software and superposed to the ligand of reference [38]. Discovery Studio Visualizer program was used to identify hydrogen bonds and other hydrophobic interactions [39].

### 2.6 *In silico* ADMET prediction

The profiling of compound pharmacokinetics is very essential in drug discovery. As of today, many online tools are available to predict the ADMET profiles of drugs based on their chemical properties [40]. In silico ADMET profiling can be useful to speed up the drug development process by limiting the number of compounds for experimental testing. In this study, physicochemical properties and pharmacokinetic parameters were evaluated using SwissADME online calculation toolkit (http://www.swissadme.ch/) [41]. Lipinski’s rule of five was taken into account to assess the ability of the compounds to be active for orally administrated drugs [42]. Other parameters such as water solubility, gastrointestinal absorption, and blood-brain barrier permeability were predicted. Pain-assay interference compounds (PAINS) are chemical molecules that often give false-positive results in high-throughput screens due to the presence of several disruptive functional groups that interact nonspecifically with various biological targets rather than selectively affecting the therapeutic target of interest [43]. Hepatotoxicity was predicted using the ProTox-II web server (https://tox-new.charite.de/protox_II/) [44].

### 2.7 Molecular dynamics simulations

AMBER16 software [45] was employed to conduct molecular dynamics simulations on the most potent compounds in complex with MAO-B. AMBER force field 14SB [46] and the general AMBER force field (GAFF2) [47] were used to parametrize the protein and the identified inhibitors, respectively. The TIP3P water model with a margin of 15.0 Å (1.5 nm) in each direction from the solute was used to construct a water-solvated cubic box. The specifics of the used MD simulations are elucidated in Ref. [48–55]. In synopsis, energy minimization was initially used on the investigated inhibitors in complex with MAO-B for 5000 steps using the combined steepest and conjugate gradient algorithms. Thereafter, the minimized systems were progressively heated from 0 k to 300 k over 50 ps. The complexes were equilibrated to a free simulation for 1000 ps. Ultimately, a production run for 100 ns was subsequently carried out utilizing an NPT ensemble at 300 K with 1.0 atm pressure. All the periodic boundary PME (Particle Mesh Ewald) simulations were conducted using the “pmemd.cuda” implementation in AMBER for GPU-accelerated simulations on the CompChem hybrid GPU/CPU cluster.

### 2.8 MM-GBSA binding energy

The molecular mechanics-generalized Born surface area (MM-GBSA) approach was applied to estimate the binding free energies (Δ*G*_binding_) of the investigated inhibitors in complex with MAO-B [56]. Thus, the total binding free energies were evaluated according to IGB value of 2. For each system, the binding free energy calculations were executed for 10,000 snapshots recorded throughout 100 ns MD simulations. For each snapshot, the MM-GBSA (Δ*G*_binding_) binding energy was calculated by the standard formula:

$$\Delta G_{\text{binding}}=G_{\text{complex}}-\left(G_{\text{inhibitor}}+G_{\text{MAO }-\text{ B}}\right)$$

## 3 Results

Developing efficient therapies against neurodegenerative diseases such as Parkinson’s disease remains a great challenge. The use of natural products has been known for a long time to offer great promise and they’re often a safer alternative compared to synthetized drugs. Currently, *in silico* studies are providing much-needed preliminary data about potential drugs, which can be a great help in conducting additional *in vitro* and *in vivo* studies [57].

### 3.1 Validation of molecular docking accuracy

**Figure 5:**
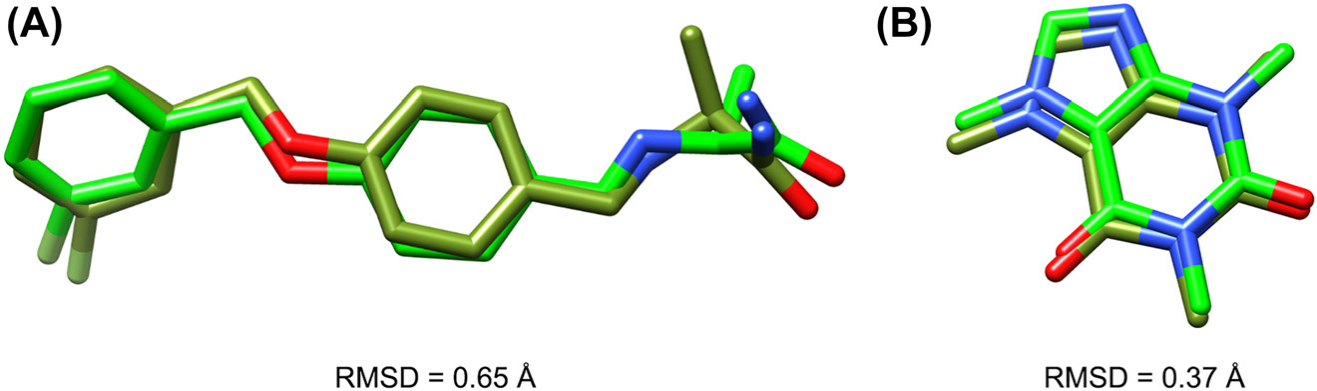
(A) Superposition and RMSD of crystal (green) and docked (olive green) structure of safinamide. (B) Superposition and RMSD of crystal (green) and docked (olive green) structure of caffeine.

Molecular docking protocol was first validated by cross-docking the co-crystallized ligands using the same parameters that were applied for the studied compounds against different crystallographic structures (PDB ID: 2V61 for MAO-B and PDB ID: 5IU4 for AA_2A_R) [13, 58]. Computed root-mean-square deviation (RMSD) was calculated by mean of superposition; the obtained values are below 2 Å which indicates a good quality of the docking program (Figure 5).

**Figure 6:**
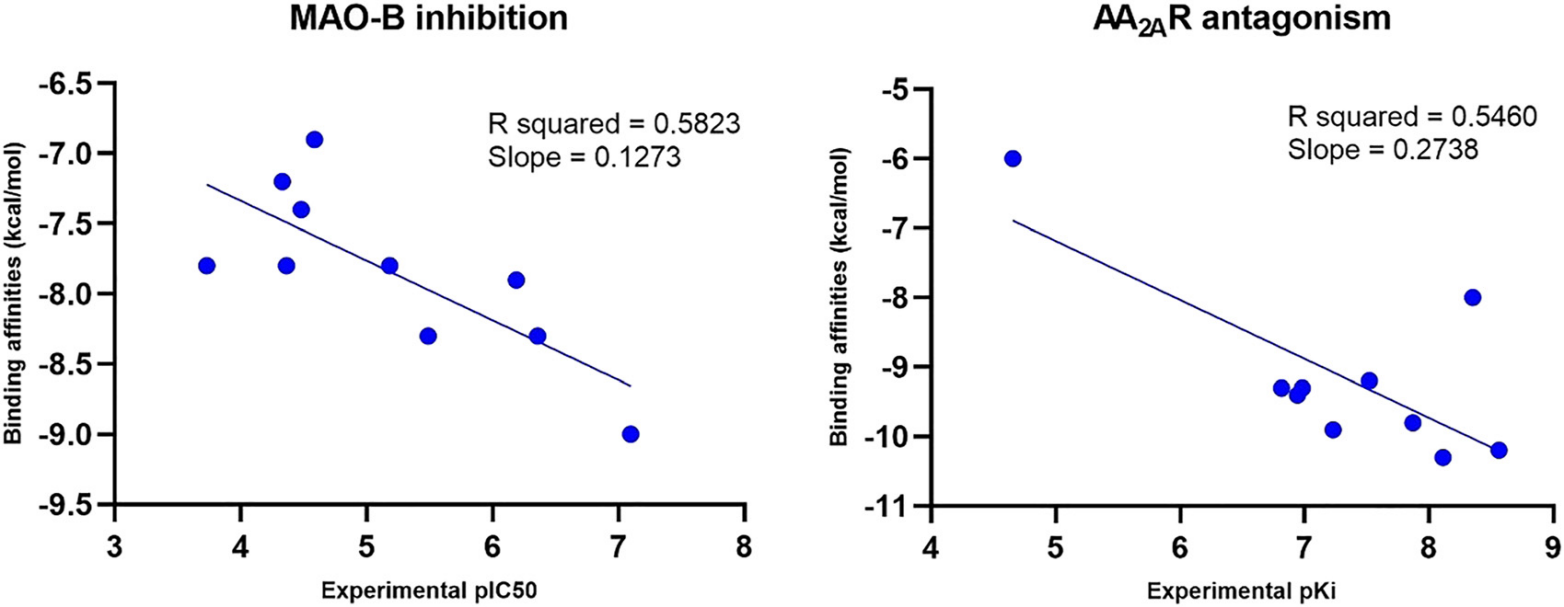
Correlation chart between molecular docking results and experimental pIC50 for MAO-B (left) and experimental pKi for AA_2A_R (right).

Additionally, molecular docking accuracy was further validated using two datasets of 10 caffeine derivatives with reported half-maximal inhibitory concentrations (IC_50_) for MAO-B and dissociation constants (Ki) for AA_2A_R respectively [24, 59]. A good correlation was established between the docking results and the experimental values for MAO-B and AA_2A_R, which confirms the reliability of the molecular docking approach to study the natural product-like caffeine derivatives with MAO-B. The correlation charts, correlation coefficients, and slopes are shown in Figure 6.

### 3.2 Natural product-like caffeine derivatives screening

In the present study, we screened 144 natural product-like caffeine derivatives against MAO-B using structure-based virtual screening. The compounds were ranked by their binding affinities (kcal/mol). The highest-ranking molecules displaying a docking score of −8.0 kcal/mol or less were further analyzed based on their interactions with the MAO-B active site cavity. Molecular docking results and protein-ligand interactions are shown in Table 1.

**Table 1:** Docking results and protein-ligand interactions between the highest-scoring compounds and MAO-B.

Compound	Chemical structure	Docking score	H bonds	Hydrophobic interactions
		(kcal/mol)		
Safinamide	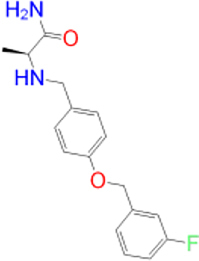	−9.9	GLN-206	TRP-119, TYR-60, LEU-164, LEU-171, TYR-326, PHE-168, GLN-206, TYR-398, TYR-435
Istradefylline	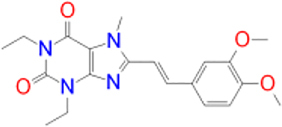	−9.3	FAD-1502	TYR-60, PRO-102, TYR-326, MET-341, LEU-328, GLN-206, ILE-199, TYR-398, TYR-435
CNP0202316	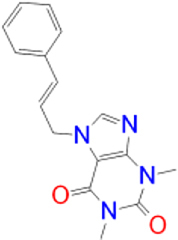	−10.1	CYS-172	TYR-60, LEU-328, TYR-326, PHE-343, TYR-398, ILE-316, LEU-171, ILE-198, ILE-199, CYS-172, PHE-168
CNP0298322	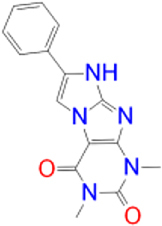	−9.8	ILE-199, TYR-435	PHE-168, ILE-164, ILE-316, ILE-198, CYS-172, TYR-398, TYR-60, PHE-343, LEU-328
CNP0369093	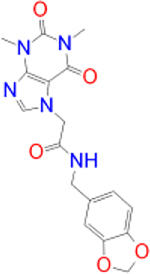	−9.8	CYS-172	ILE-199, LEU-171, ILE-199, ILE-316, TYR-326, FAD-1502, TYR-60, PHE-343
CNP0365210	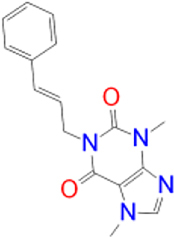	−9.7	TYR-435	ILE-198, CYS-172, LEU-171, ILE-316, ILE-199, TYR-326, PHE-343, LEU-328, TYR-398
CNP0366822	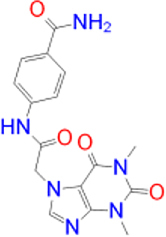	−9.7	CYS-172	TYR-60, TYR-398, TYR-435, PHE-343, LEU-171, TYR-326, ILE-199, ILE-316, ILE-198
CNP0352436	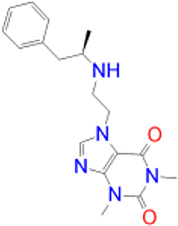	−9.6	TYR-326	TYR-60, LEU-171, ILE-316, TYR-398, TYR-435, CYS-172, ILE-199
CNP0349562	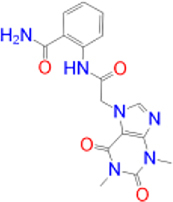	−9.5	TYR-435, FAD-1502	ILE-199, ILE-316, LEU-171, PHE-168, TYR-326, LEU-328, PHE-343, TYR-60
CNP0006822	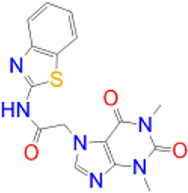	−9.4	CYS-172, TYR-435, PRO-102, FAD-1502	TYR-60, PHE-343, LEU-328, LEU-171, ILE-199, TYR-398, GLN-206
CNP0074857	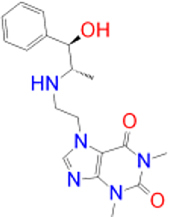	−9.2	GLN-206, LEU-171	TRP-119, LEU-167, TYR-398, TYR-326, ILE-199, ILE-316, PHE-168
CNP0390050	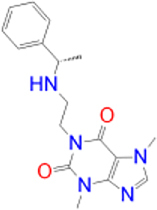	−9.1	GLN-206, TYR-435, LEU-171	TRP-119, ILE-199, ILE-316, CYS-172, PHE-343, TYR-60, TYR-398, PHE-168
CNP0089299	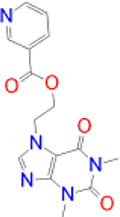	−9.0	GLN-206, TYR-435, FAD-1502	TYR-60, TYR-326, ILE-316, LEU-164, TRP-119, LEU-328, PHE-343, LEU-171, TYR-398
CNP0370968	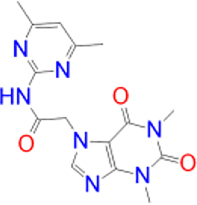	−9.0	GLN-206, TYR-435, FAD-1502	PHE-343, TYR-60, TYR-326, ILE-316, LEU-164, LEU-328, LEU-171, CYS-172
CNP0338201	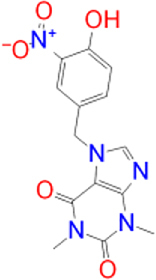	−8.9	ILE-198, TYR-435, FAD-1502	ILE-199, TYR-326, TYR-398, LEU-328, ILE-316, LEU-164, TYR-60, PHE-343, LEU-171
CNP0074614	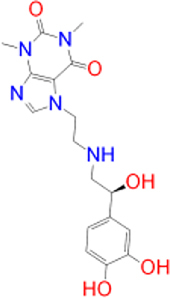	−8.8	LEU-164, TYR-326, TYR-435, FAD-1502	ILE-198, TYR-398, LEU-171, TYR-60, PHE-343, LEU-328, CYS-172, ILE-199, ILE-316, LEU-167
CNP0212890	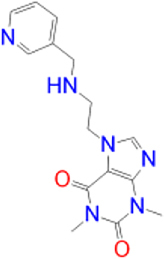	−8.7	ILE-199, FAD-1502	TYR-60, TYR-435, PHE-343, LEU-171, TYR-398, CYS-172, ILE-198
CNP0010096	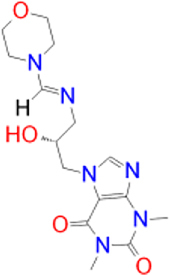	−8.4	CYS-172	ILE-316, LEU-171, TYR-326, ILE-199, PHE-168, TYR-398, TYR-326, LEU-167
CNP0276217	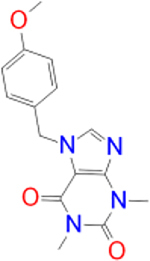	−8.3	−	LEU-167, TYR-326, LEU-164, ILE-316, PRO-102, ILE-199, LEU-171, CYS-172, PHE-343, TYR-398
CNP0370378	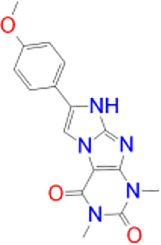	−8.2	−	TRP-119, ILE-316, TYR-326, TYR-398, TYR-435, LEU-171, ILE-199, FAD-1502
CNP0224039	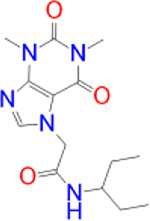	−8.2	TYR-435, FAD-1502	LEU-171, TYR-326, TYR-60, LEU-326, PHE-168
CNP0383986	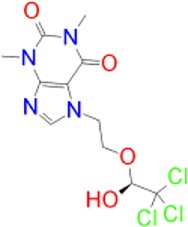	−8.0	CYS-172	TYR-60, LEU-328, PHE-343, TYR-435, ILE-316

### 3.3 *In silico* ADMET prediction results

*In silico* pharmacokinetics, toxicity, and drug-likeness prediction results are shown in Table 2. All compounds were predicted as either soluble or highly soluble. Furthermore, most of the molecules are showing high gastro-intestinal absorption which indicates a good oral bioavailability. However, the blood-brain barrier permeability parameter revealed only two compounds besides safinamide that may readily cross the blood-brain barrier and act on the central nervous system. Moreover, all compounds, excluding safinamide, were identified as non-inhibitors of CYP2D6, which is particularly necessary for drugs acting on the brain since the expression of CYP2D6 is higher in the brain and is involved in metabolizing endogenous neural compounds that suggest its neuroprotective effects [60]. Moreover, the inhibition of CYP enzymes can decrease drug efficacy leading to therapeutic failure or increased drug side effects and toxicity [61–63]. Organ toxicity predicted using the ProTox-II webserver revealed that all the compounds are safe for the liver and do not disrupt its normal function. Physicochemical properties profiling of the selected compounds revealed that all the compounds are drug-like according to Lipinski’s rule of five. Finally, Pain-assay interference compounds (PAINS) alerts calculations indicated that all the compounds do not contain any disruptive functional groups except CNP0074614 displaying one PAINS alert due to the catecholamine group.

**Table 2:** Pharmacokinetics, toxicity prediction and drug likeness of the selected compounds.

Compound	Water	GI absorption	BBB	CYP2D6	Hepatotoxicity	Lipinski	PAINS
	solubility			inhibitor		violation	alert
Safinamide	−3.04	High	Yes	Yes	Inactive	0	0
Istradefylline	−3.83	High	No	No	Inactive	0	0
CNP0202316	−3.28	High	Yes	No	Inactive	0	0
CNP0298322	−3.67	High	No	No	Inactive	0	0
CNP0369093	−3.31	High	No	No	Inactive	0	0
CNP0365210	−3.28	High	Yes	No	Inactive	0	0
CNP0366822	−2.75	Low	No	No	Inactive	0	0
CNP0352436	−4.46	High	No	No	Inactive	0	0
CNP0349562	−2.75	Low	No	No	Inactive	0	0
CNP0006822	−3.72	High	No	No	Inactive	0	0
CNP0074857	−3.52	High	No	No	Inactive	0	0
CNP0390050	−4.07	High	No	No	Inactive	0	0
CNP0089299	−2.90	High	No	No	Inactive	0	0
CNP0370968	−3.20	High	No	No	Inactive	0	0
CNP0338201	−1.98	High	No	No	Inactive	0	0
CNP0074614	−2.33	Low	No	No	Inactive	0	1
CNP0212890	−3.67	High	No	No	Inactive	0	0
CNP0010096	−0.80	Low	No	No	Inactive	0	0
CNP0276217	−3.32	High	No	No	Inactive	0	0
CNP0370378	−3.79	High	No	No	Inactive	0	0
CNP0224039	−2.30	High	No	No	Inactive	0	0
CNP0383986	−2.15	High	No	No	Inactive	0	0

**Water solubility,** insoluble < −10 < poorly < −6 < moderately < −4 < soluble < −2 < very < 0 < highly; **GI absorption,** gastrointestinal absorption; **BBB,** blood-brain barrier permeability; **CYP2D6 inhibitor,** Likeliness of a drug to act as inhibitor of cytochrome P450 CYP2D6; **Hepatotoxicity,** prediction of drug-induced liver injury; **Lipinski violation,** number of violations to the rule of five (log *P*_o/w_ ≤ 5; MW ≤ 500 g/mol; HBA ≤ 10; HBD ≤ 5; RB ≤ 10); **PAINS alert,** number of disruptive functional groups shared by many PAINS (Pan-assay interference compounds).

### 3.4 Interaction analysis of lead compounds with MAO-B and AA_2A_R

**Figure 7:**
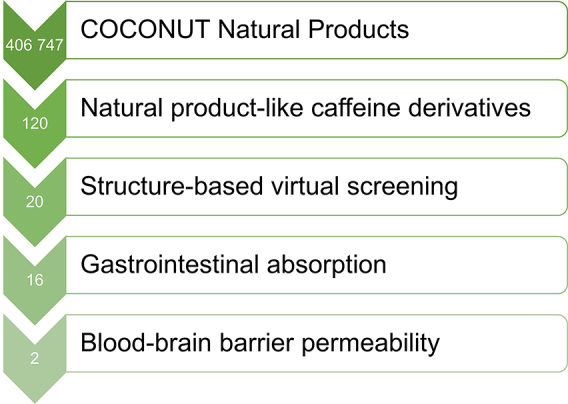
Step-wise structure and ADMET-based screening of the selected natural product-like caffeine derivatives.

**Figure 8:**
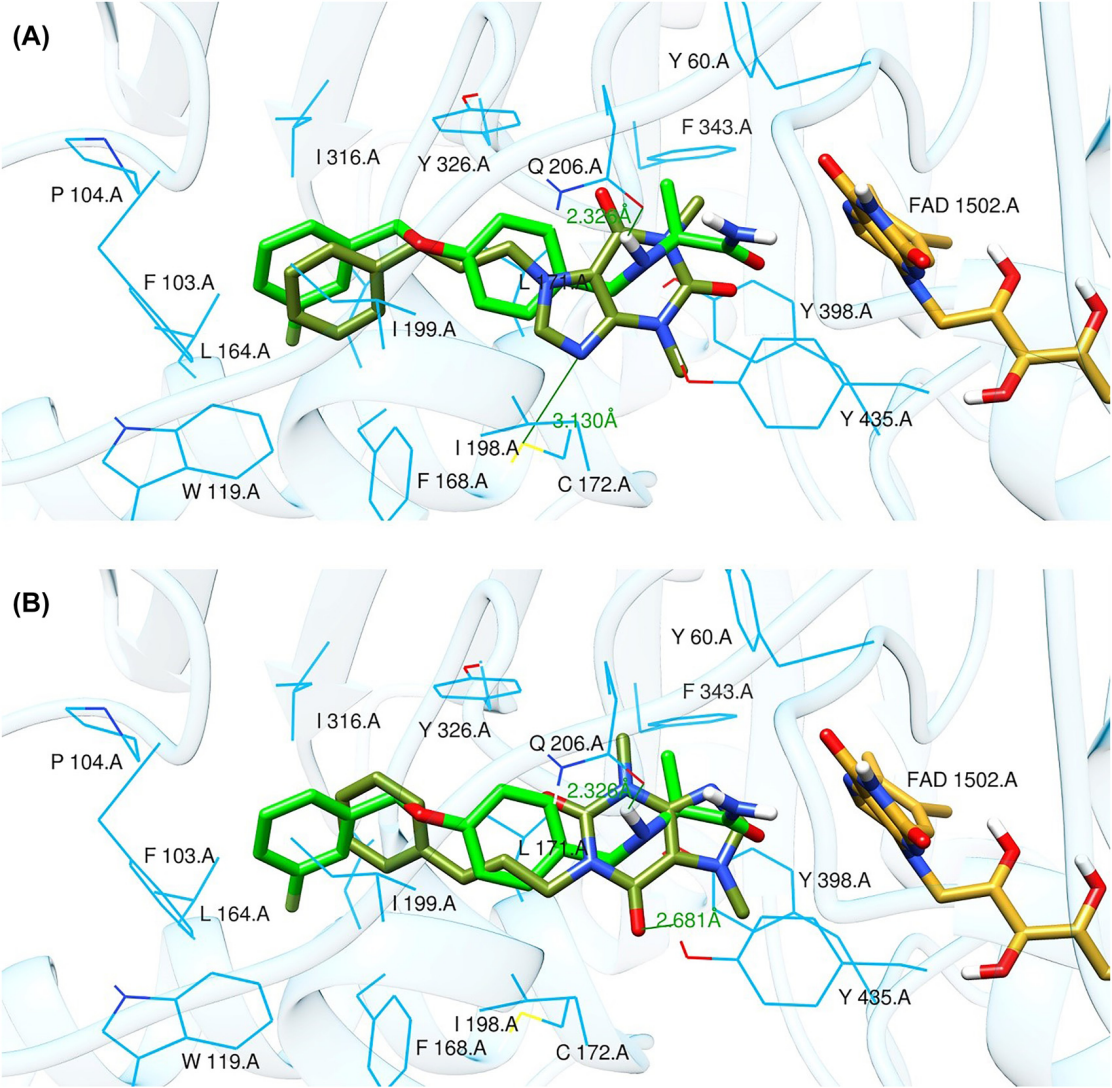
Binding poses of selected lead compounds: CNP0202316 (A) and CNP0365210 (B) (shown in olive green color) with MAO-B and superposed to safinamide (shown in green color).

According to the molecular docking and ADMET-based screening as summarized in Figure 7, two compounds were identified as potential drug candidates that possess the desired pharmacokinetics properties for drugs acting on the central nervous system: CNP0202316 and CNP0365210 superposed to the reference inhibitor, safinamide in complex with MAO-B are illustrated in Figure 8. The binding scores of these molecules were −10.1 and −9.7 kcal/mol for MAO-B respectively and are comparable to the reference inhibitor, safinamide which displayed a binding score of −9.9 kcal/mol. However, molecular docking of istradefylline revealed a low binding affinity (−9.3 kcal/mol) compared to the aforementioned compounds.

Safinamide was found to interact through hydrogen bonding with GLN-206 which is known to be a hydrogen bond acceptor for the majority of MAO-B inhibitors and is by the literature [13]. Structural analysis shows that both compounds share a phenyl ring linked to the caffeine scaffold with a pentane group. The phenyl ring seems to be favorable for the stability of the ligands within the MAO-B active site by establishing numerous hydrophobic interactions with the nearby residues of the entrance cavity. Meanwhile, the caffeine scaffold is directed towards the FAD cofactor and interacts with CYS-172 in CNP0202316 and TYR-435 of the aromatic cage in CNP0365210 through hydrogen bonding.

It has been shown in previous studies that hydrophobic interactions through the phenyl ring are vital for establishing MAO-B binding and are more favorable than all other interactions such as hydrogen or halogen bonds [64]. However, CNP0202316 where the phenyl ring is placed at C7 of the xanthine core seems to be more stable than the second compound implying that the presence of hydrophilic interaction with CYS-172 contributes more to the anchoring and the stability of this compound in the active site cavity of MAO-B.

Alternatively, the selected compounds were analyzed through molecular docking studies with AA_2A_R to assess their binding affinities with the AA_2A_R active site and compare their binding conformations to istradefylline. Molecular docking results and protein-ligand interactions are shown in Table 3.

**Table 3:** Molecular docking results and protein-ligand interactions of selected lead compounds with AA_2A_R.

Compound	Chemical structure	Docking score	H bonds	Hydrophobic interactions
		(kcal/mol)		
Istradefylline	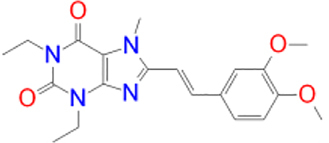	−8.0	ASN-253	ALA-81, PHE-168, GLU-169, MET-174, LEU-249, TYR-271
CNP0202316	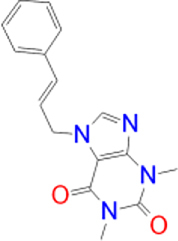	−8.7	ASN-253	LEU-85, ILE-66, LEU-167, PHE-168, GLU-169, TRP-246, LEU-249, ILE-274, LEU-267, TYR-271
CNP0365210	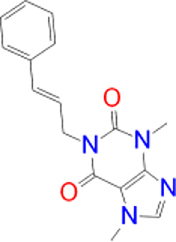	−8.0	ASN-253	LEU-85, ILE-66, PHE-168, GLU-169, TRP-246, LEU-267, TYR-271

**Figure 9:**
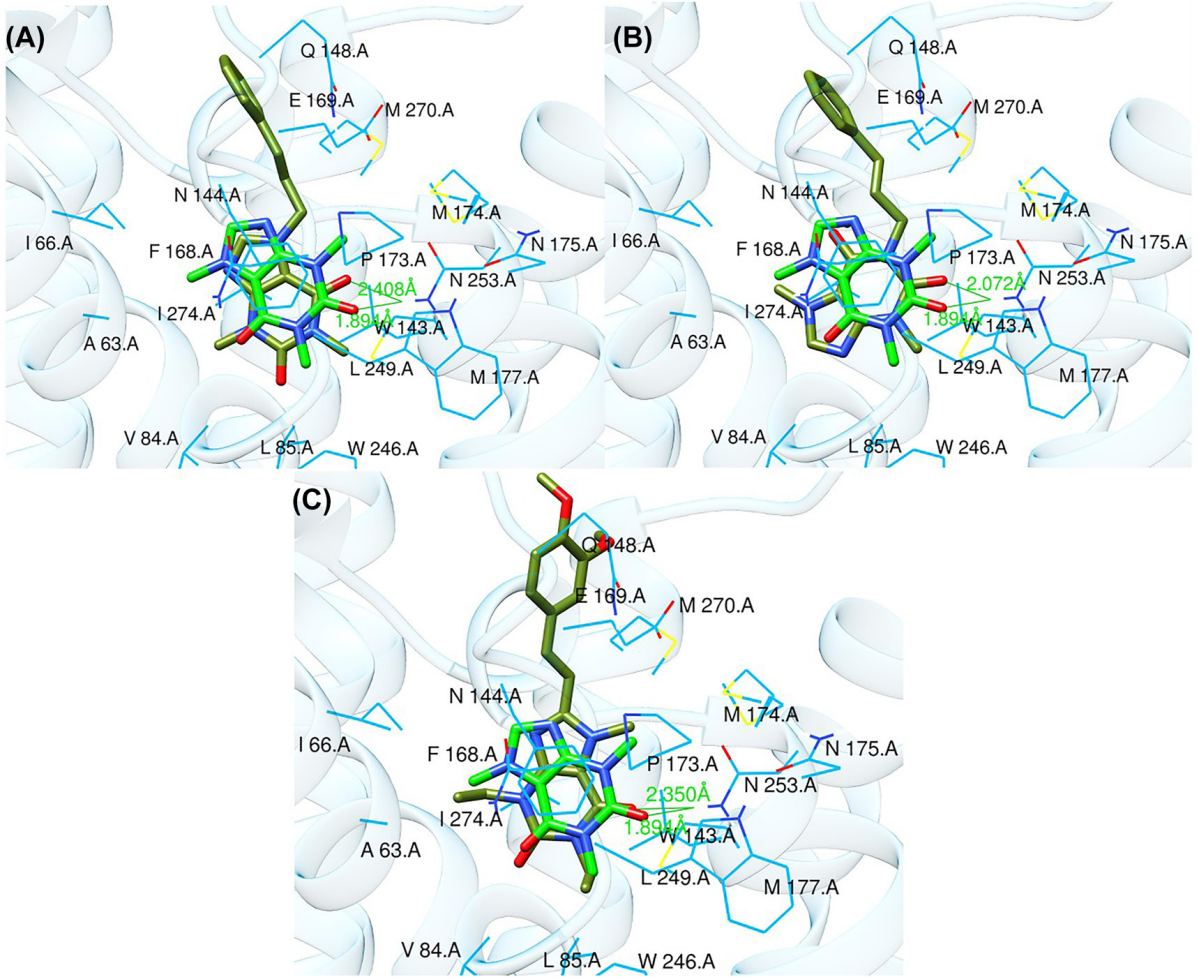
Binding poses of CNP0202316 (A), CNP0365210 (B), and istradefylline (C) (shown in olive green color) with AA_2A_R and superposed to the crystal structure of caffeine (shown in green color).

The binding poses of the selected compounds and istradefylline were superposed to the co-crystallized structure of caffeine (Figure 9). The structural analysis indicates that both compounds were able to interact with a key polar residue, ASN-253 through the formation of hydrophilic hydrogen bonds, similarly to istradefylline and other potent AA_2A_R antagonists [65]. A large network of hydrophobic interactions was also observed, where key residues namely PHE-168 and GLU-169 were found to make a consistent appearance. Since all compounds share the same core that characterizes the caffeine molecule, this would entail a somewhat similar disposition inside the binding pocket. This holds especially for CNP0202316 where the xanthine core was found to be positioned similarly to istradefylline. Moreover, the propylbenzene moiety at position C7 in CNP0202316 might be more favorable to the hydrophobic pocket of the receptor suggesting its affinity potential which may be on par or better than istradefylline.

### 3.5 Molecular dynamics simulations and binding energy calculations

**Figure 10:**
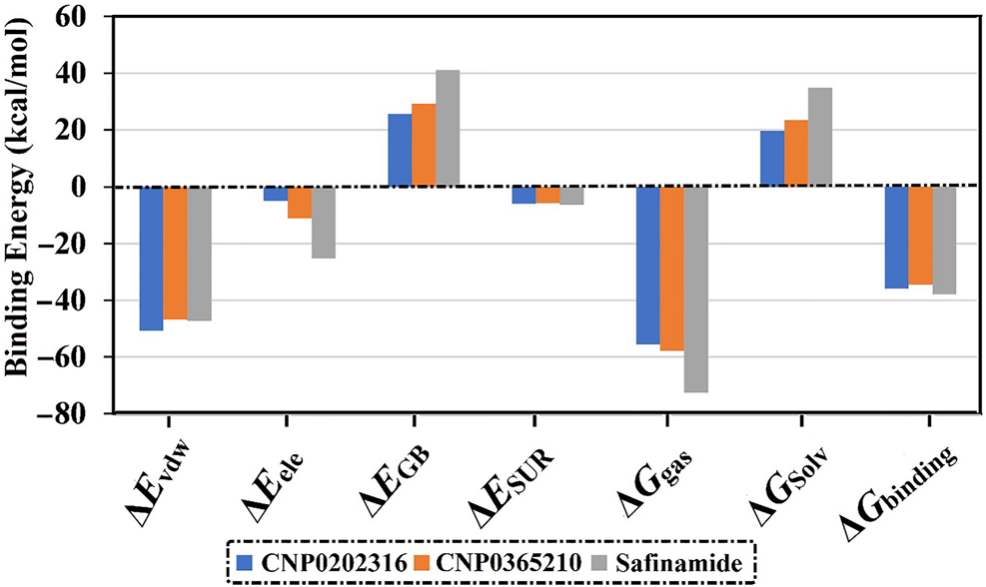
Decomposition of MM-GBSA binding energies for the investigated inhibitors in complex with MAO-B throughout 100  ns MD simulations.

Molecular dynamics (MD) simulations were applied to probe the stability of the selected ligand-protein complexes, structural specifics, conformational flexibilities, and realize reliable inhibitor-enzyme binding affinities [66, 67]. The most promising compounds in complex with the MAO-B enzyme were further inspected via MD throughout 100 ns simulation time. According to the gathered inhibitor-enzyme snapshots over the production run of 100 ns, the MM-GBSA approach was used to calculate the binding free energies (Δ*G*_binding_) and are illustrated in Figure 10. From the data in Figure 10, it is apparent that the CNP0202316 and CNP0365210 demonstrated auspicious binding affinities with values of −36.7 and −34.5 kcal/mol, respectively, and are comparable to the reference inhibitor, safinamide (Δ*G*_binding_ = −37.9 kcal/mol). The comparison of safinamide with CNP0202316 and CNP0365210 unveiled competing for binding affinities proposing the *in silico* prospectivity of the two molecules as MAO-B inhibitors.

The calculated MM-GBSA binding energies were then decomposed into separate components to recognize the vigor in the binding of MAO-B with CNP0202316, CNP0365210, and safinamide (Figure 10). The van der Waals (Δ*E*_vdw_) energy was a considerable contributor to CNP0202316, CNP0365210, and safinamide-MAO-B binding affinities with average values of −50.9, −46.8, and −47.3 kcal/mol, respectively. Δ*E*_ele_ was effectual with average values of −5.2, −11.1, and −25.3 kcal/mol for the CNP0202316, CNP0365210, and safinamide-MAO-B binding affinities, respectively.

**Figure 11:**
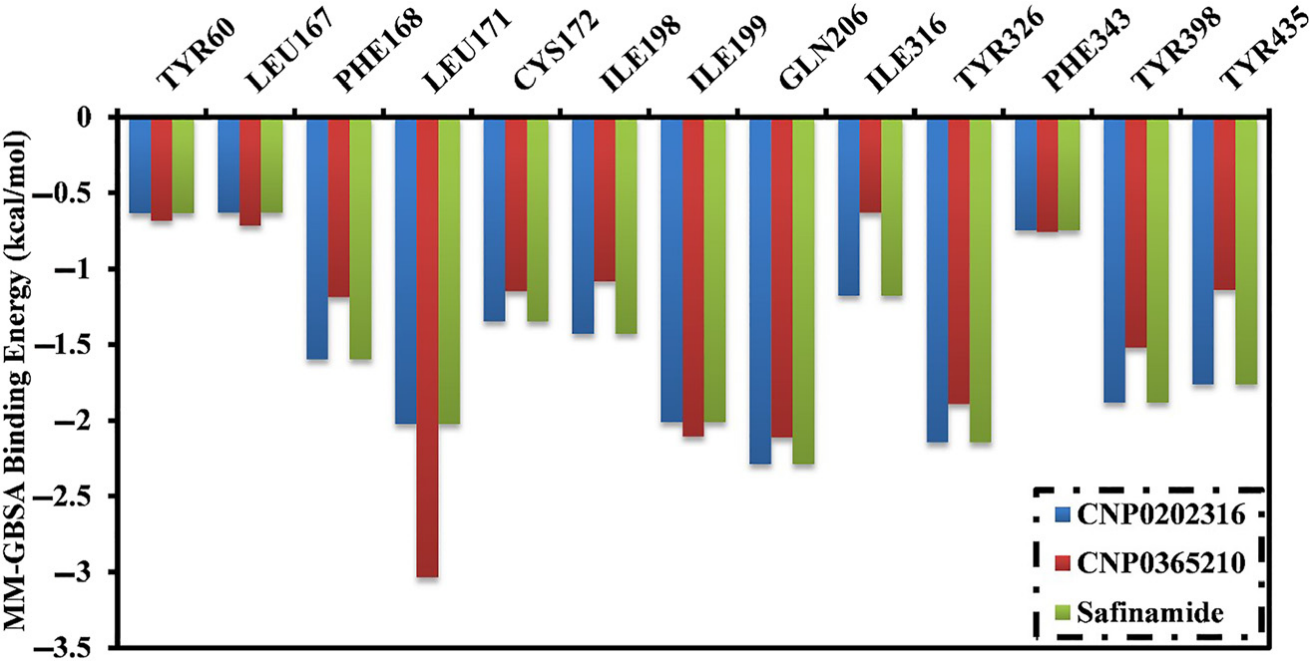
Energy contributions (kcal/mol) for MAO-B amino acid residues to the binding free energy of CNP0202316, CNP0365210, and safinamide.

The binding energies of CNP0202316, CNP0365210, and safinamide in complex with MAO-B were further decomposed at the per-residue level, and the amino acid residues with free energy contribution <−0.50 kcal/mol were depicted (Figure 11). LEU-171, GLN-206, and TYR-326 in the MAO-B complex appropriately share with CNP0202316, CNP0365210, and safinamide. There was significant participation by LEU-171 to the total binding free energy with values of −2.0, −3.0, and −2.2 kcal/mol for CNP0202316-, CNP0365210- and safinamide-MAO-B complexes, respectively.

### 3.6 Post-MD simulations analysis

Molecular docking calculations, and MD simulations combined with MM-GBSA binding energy calculations, unveiled the most potent molecules as potential MAO-B inhibitors. The MD-based analysis could be required to demonstrate structural and energetic stabilities for the scrutinized inhibitors in complex with MAO-B. The structural and energetical analysis included root-mean-square deviation (RMSD), Center-of-Mass (CoM) distance, and binding energy per-frame.

#### 3.6.1 Root-mean-square deviation (RMSD)

**Figure 12:**
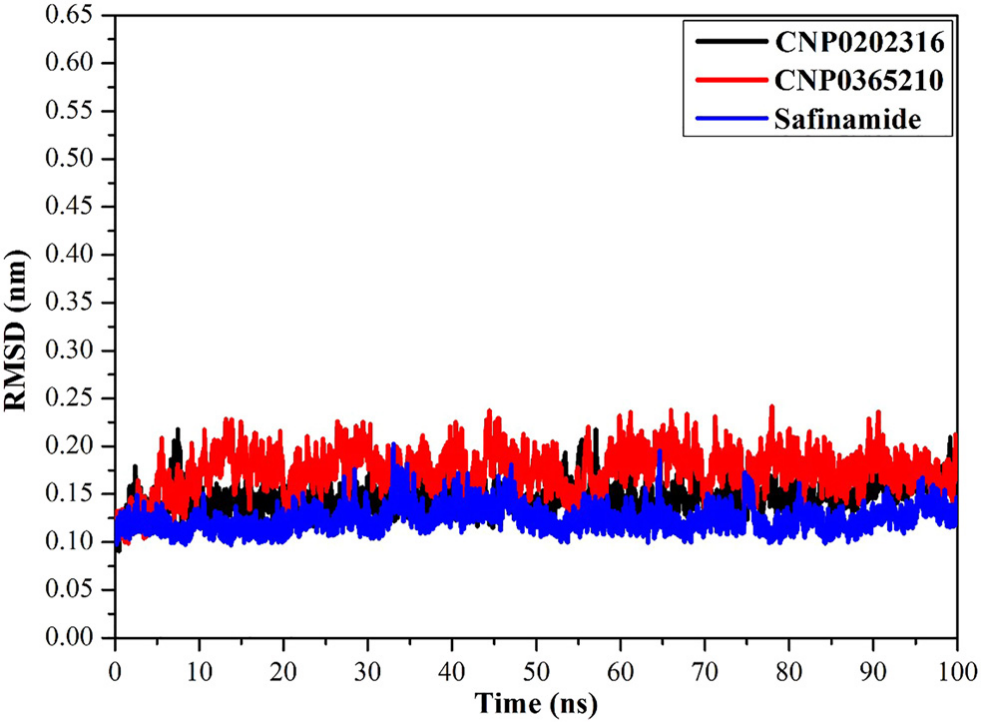
Root-mean-square deviation (RMSD) of the backbone atoms from the starting structure of CNP0202316 (in black), CNP0365210 (in red), and safinamide (in blue) with MAO-B during the 100 ns MD simulations.

The root-mean-square deviation (RMSD) values of the backbone atoms within the whole complex throughout the simulation time were estimated to monitor the structural stability of the CNP0202316, CNP0365210, and safinamide in complex with MAO-B. The RMSD of the backbone atoms as a function of time following the initial structure of the three investigated systems is displayed in Figure 12. The platform in RMSD curves emphasizes that all three inspected systems attain an equilibrium within 1000–10,000 ps throughout MD simulations, exposing that the three investigated systems are converged over the simulation window. These findings suggest that all the compounds are tightly bound and not influenced by the topology of the protein. This is especially true for CNP0202316, which displayed very slight deviations somewhat similar to safinamide suggesting its high stability compared to CNP0365210.

#### 3.6.2 Center-of-mass distance

**Figure 13:**
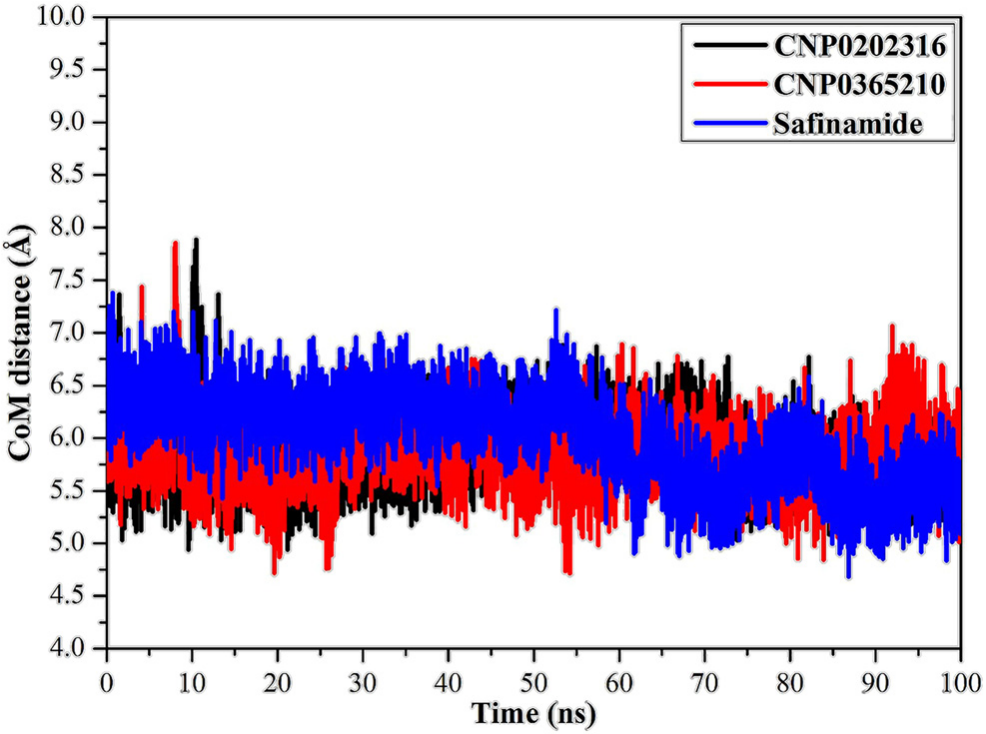
Center-of-mass (CoM) distances (in Å) between CNP0202316 (in black), CNP0365210 (in red), and safinamide (in blue) and TYR324 of MAO-B throughout a 100 ns MD simulation.

To get a more in-depth insight into the stability of the selected compounds throughout the MD simulation time, center-of-mass (CoM) distances were evaluated (Figure 13). The most interesting aspect of this graph is that CoM distances were consistent for the CNP0202316- and CNP0365210 in complex with MAO-B compared to safinamide-MAO-B complex, with average values of 5.7, 5.8, and 6.1 Å, respectively. The most obvious finding to emerge from this analysis is that CNP0202316 and CNP0365210 bound more tightly to the MAO-B complex than the reference inhibitor, safinamide.

#### 3.6.3 Binding energy per frame

**Figure 14:**
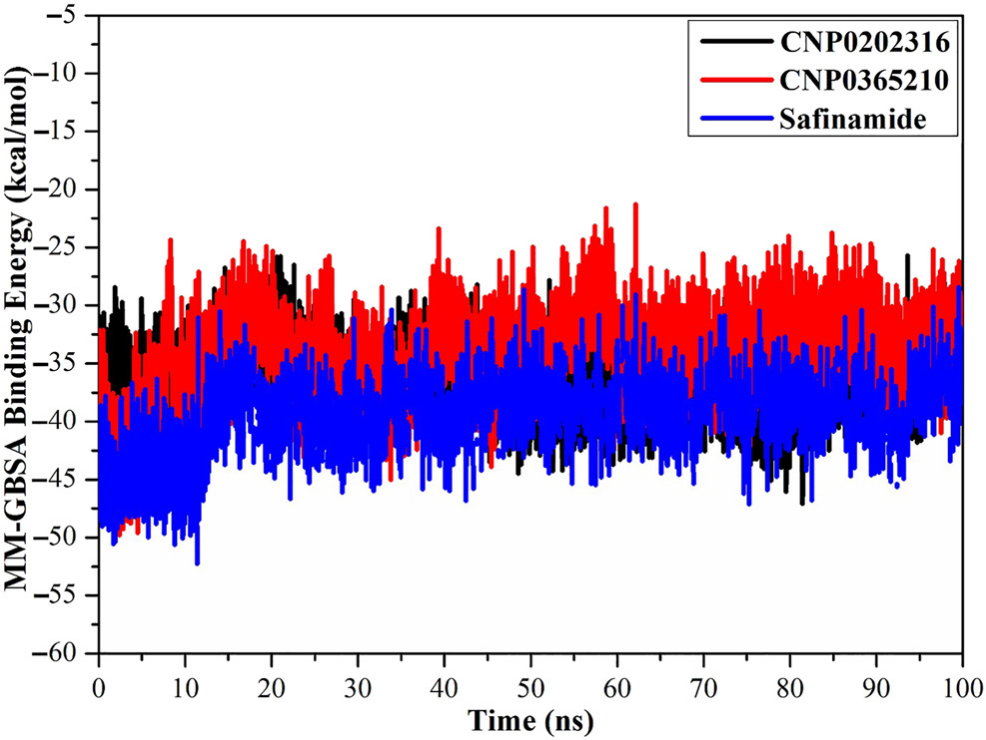
Estimated MM-GBSA binding energy per frame for CNP0202316 (in black), CNP0365210 (in red), and safinamide (in blue) with MAO-B over a 100 ns MD simulation.

The comprehensive structural stability of CNP0202316, CNP0365210, and safinamide complexed with MAO-B was evaluated throughout a 100 ns MD simulation via inspecting the correlation between the binding energy per frame and time (Figure 14). Overall stabilities for CNP0202316, CNP0365210, and safinamide were noticed with average binding energies (Δ*G*_binding_) of −36.6, −34.5, and −37.9 kcal/mol, respectively. Based on this analysis, all investigated complexes preserved their stability over the 100 ns MD simulations.

## 4 Discussion

The results obtained from the molecular docking study with MAO-B were further analyzed through molecular dynamics simulations and binding free energy calculations. MM-GBSA binding energies further confirmed that van der Waals (Δ*E*_vdw_) energy is a considerable contributor to the stability of MAO-B inhibitors. This finding confirms that hydrophobic interactions outweigh any other interactions in terms of MAO-B inhibition, and is also supported by the literature [65]. Post-MD simulations analysis confirmed the stability of the three compounds and revealed that all the complexes achieve equilibrium within 1–10 ns throughout the simulation time. These results suggest that the proposed natural products may be on par or better than the inhibitor of reference, safinamide regarding MAO-B inhibition. The key residues involved in MAO-B inhibition were found to be LEU-171, ILE-199, TYR-326, and GLN-206, these residues contribute the most to the stability of the inhibitors when bound to MAO-B. LEU-171, ILE-199, and TYR-326 are specific residues to MAO-B isoform, they are located in the entrance cavity and play a role in substrate and inhibitor specificity [68, 69]. Meanwhile, GLN-206 is recognized as a hydrogen bond acceptor for most MAO-B inhibitors and is responsible for their stability in the substrate cavity [17]. Moreover, the identified compounds may confer neuroprotective properties linked to the xanthine core as reported in the literature [70]. For MAO-B, the orientation of the phenyl ring linked to the xanthine core was found to be similar to safinamide especially in CNP0202316, and is favorable to the entrance hydrophobic cavity. On the other hand, this molecule adopted a similar conformation to istradefylline when bound to AA_2A_R. The propylbenzene moiety attached to the imidazole of the caffeine in CNP0202316 at position C7 is more favorable to the hydrophobic pocket of AA_2A_R, whereas the oxygen atom of the xanthine core maintained a hydrogen bond with ASN-253 that is deemed crucial to the binding of most AA_2A_R antagonists [71]. Thus, the identified compounds might offer a dual-target activity in the context of a polypharmacological approach and might represent a more efficient alternative for treating and slowing down the neuronal damage in Parkinson’s disease patients.

## 5 Conclusions

The present study aimed to find novel natural product-like caffeine derivatives as potential dual MAO-B inhibitors/AA_2A_R antagonists. Structure-based virtual screening and ADMET analysis revealed two natural products that fulfill the requirements for drugs acting on the brain. The selected compounds in complex with MAO-B were subject to molecular dynamics simulations to assess their stability over the simulation time along with the inhibitor of reference, safinamide. Our findings show that the presence of the phenyl ring in the selected compounds is crucial for the ligands to fill the long-shaped cavity of the MAO-B active site and is a major contributor to various van der Waals interactions responsible for the stability and the tight-binding of these compounds to MAO-B. Similarly, the propylbenzene moiety was found to be more favorable for the hydrophobic pocket of AA_2A_R especially when linked at position C7 of the xanthine core which allowed the caffeine core to adopt a similar conformation to istradefylline suggesting the dual-target properties of the identified natural products. In conclusion, the structure-based virtual screening helped provide valuable insight on the studied natural product-like caffeine derivatives and our findings may attract more focus for the development of novel antiparkinsonian drugs with dual-targeting properties. However, *in vitro* experiments such as bioactivity assays for MAO-B and AA_2A_R, membrane permeability and cell viability assays remains necessary to further validate these findings.
